# Localized Induction of Gene Expression in Embryonic Stem Cell Aggregates Using Holographic Optical Tweezers to Create Biochemical Gradients

**DOI:** 10.1007/s40883-019-00114-5

**Published:** 2019-08-26

**Authors:** Glen R Kirkham, James Ware, Thomas Upton, Stephanie Allen, Kevin M Shakesheff, Lee DK Buttery

**Affiliations:** 1 College of Science and Technology, Nottingham Trent University, Nottingham, NG11 8NS UK; 2 School of Pharmacy, University of Nottingham, Nottingham, NG7 2RD UK

**Keywords:** Stem cells, Embryonic stem cells, In vitro model, Optical tweezers, Stra 8, Retinoic acid, Embryoid bodies, HOTs

## Abstract

Three-dimensional (3D) cell models that mimic the structure and function of native tissues are enabling more detailed study of physiological and pathological mechanisms in vitro. We have previously demonstrated the ability to build and manipulate 3D multicellular microscopic structures using holographic optical tweezers (HOTs). Here, we show the construction of a precisely patterned 3D microenvironment and biochemical gradient model consisting of mouse embryoid bodies (mEBs) and polymer microparticles loaded with retinoic acid (RA), embedded in a hydrogel. We demonstrate discrete, zonal expression of the RA-inducible protein Stra8 within mEBs in response to release of RA from polymer microparticles, corresponding directly to the defined 3D positioning of the microparticles using HOTs. These results demonstrate the ability of this technology to create chemical microgradients at definable length scales and to elicit, with fidelity and precision, specific biological responses. This technique can be used in the study of in vitro microenvironments to enable new insights on 3D cell models, their cellular assembly, and the delivery of drug or biochemical molecules for engineering and interrogation of functional and morphogenic responses**.**

**Electronic supplementary material:**

The online version of this article (10.1007/s40883-019-00114-5) contains supplementary material, which is available to authorized users.

**Figure:**
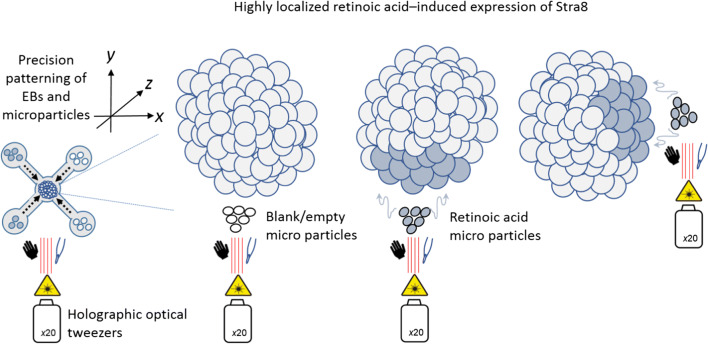
Graphical abstract

## Introduction

Our ability to understand the complex biology and physiology of cells and tissues is being advanced through innovative approaches to reproduce their multicellular three-dimensional (3D) interactions, structure, and function in vitro. With the relatively simple approach of culturing different cell types, together with specific signaling factors and natural or synthetic scaffolds, cells can be encouraged to self-assemble and form organized 3D cellular structures in vitro that mimic native tissues. Organoids and organ-on-chip technologies are now enabling investigation of specific developmental, physiological, and disease processes and to dissect the effects and mechanisms of biochemical factors or drug molecules in a controlled manner [1–8].

The level of precision and accuracy of control of cell interactions and delivery of bio-instructive signals that can be achieved is varied among these technologies, and the ability to work interactively and precisely at the scale of individual cells and their microenvironment is a particular challenge [9]. Meeting this challenge, we have established the use of holographic optical tweezers to enable simultaneous imaging and micromanipulation of multiple cells, as well polymer microparticles within 3D culture environments [10]. These can be precisely positioned and assembled, within a matter of minutes, into predetermined 3D microtissue structures, with accuracy of control from micron- to millimeter-length scales (10).

In this study, we use HOTs to create biochemical gradients within 3D microenvironments by precise positioning of microparticles loaded with a biochemical factor and induction of highly localized responses in mouse embryoid bodies (EBs). The formation of EBs from embryonic stem (ES) cells has been used extensively as a model to investigate in vitro differentiation of particular cell types in response to specific biochemical factors [11–15]. Various approaches have been used to stimulate differentiation, including simply adding biochemical factors to the culture medium [11–13] through to aggregating ES cells together with microparticles doped with biochemical factors [14, 15]. While such studies demonstrate directed and localized differentiation of ES cells and EBs in response to bio-instructive signals, the results are often highly variable, with minimal predictable and repeatable targeted control of differentiation within defined cell populations or locations within the 3D cellular aggregates.

To demonstrate the potential of HOTs to achieve precision delivery of bio-instructive signals with concomitant biological responses at defined locations within EBs we have used a robust and highly sensitive retinoic acid (RA) inducible genetic system. Recent work has shown that mES cells are sensitive to low concentrations of RA resulting in marked increases of two RA responsive elements (RAREs), stimulated by RA gene 8 (Stra8) and deleted in azoospermia-like (Dazl) [16, 17]. The expression of these two RAREs is tightly linked to RA concentration and up to 800-fold increases have been reported after just 24 h of exposure [16, 17]. This presents a robust model to demonstrate highly localized release of RA and expression of Stra8.

In this study, we show the formulation of RA encapsulated microparticles with controlled release profiles, together with an approach to enable selection of microparticles with high RA loading efficiency. Subsequently, we show the precise positioning of small numbers of RA encapsulated microparticles to defined X, Y, Z locations and distances around mEBs and the resulting highly localized induced expression of Stra8, correlating directly with the positioning of RA release source.

### Lay Summary (98/100)

Advances in growing cells in a culture dish and forming organized 3D cellular structures that mimic tissues in our body are greatly improving the study of biological processes in health and disease. Using an instrument called holographic optical tweezers, we have shown how a non-damaging light source can literally work as a pair of microscopic tweezers. We demonstrate the ability to interactively and precisely build microscopic tissues and to deliver biochemical or drug molecules and induce highly localized responses in the cells. This technology has great potential for building accurate tissue models to test and develop new drugs.

### Future Work (50/50)

To build more complex 3D models of biochemical gradients, including localized delivery of single or multiple factors and dynamic monitoring of localized release kinetics and cell responses. Recent studies on spontaneous in vitro assembly of embryo-like structures present a robust model to build localized biochemical gradients and interrogate developmental processes.

## Methods

### Holographic Optical Tweezer (HOT) Systems

Experiments were performed with the HOTs instrument as described previously [10] and a commercially available portable system (CUBE; Meadowlark Optics, USA). Both systems are based on an infra-red laser for bio-applications. All software-based control functions were programmed using LabVIEW software (National Instruments) as described previously [10]. The CUBE system was housed in a standard cell culture incubator (37 °C, 5% CO_2_) facilitating HOTs controlled patterning of cells and microparticles under standard culture conditions.

### Mouse Embryonic Stem (mES) Cell Culture

The mES cell line (denoted CGR8) [18] was cultured on 0.1% (*v*/*v*) gelatin solution. Medium consisted of Dulbecco’s modified Eagle’s medium (DMEM) (Life Technologies) supplemented with 10% (*v*/*v*) fetal bovine serum (FBS) (Sigma-Aldrich), 2 mM l-glutamine (Life Technologies), 0.1 μM 2-mercaptoethanol (Sigma-Aldrich), 50 μg/mL penicillin/streptomycin (Life Technologies), and 5000 U/mL leukemia inhibitory factor (LIF) (Calbiochem).

### Formation of Embryoid Bodies (mEBs) by Hanging Drop

Prior to aggregation, mES cells were transiently serum started by culturing in mES cell culture medium without FCS for 24 h to minimize any RA-induced responses before the controlled release experiments. Uniformly sized cell aggregates, mES cells were created by the hanging drop method [13]. Cell suspensions were diluted to 2 × 10^4^ cells/mL in the same medium as above without LIF and then, using a multi-flow pipette (8 tips), 25-μL volumes were deposited onto the underside of a 60 mm Petri dish lid to form eight rows. The resulting 64 droplets containing mES cells were then inverted and the lid is placed on the Petri dish containing PBS. The droplets containing roughly 500 cells were cultured for 24 h and the resulting cell aggregates were collected and suspended in a pre-gel GelMA solution ready for patterning on the HOTS system.

### Immunohistochemical Staining of Stra8 in mES Cells and mEBs

Dose–response effects of RA on expression of Stra8 were initially assessed by adding RA directly to culture medium and culturing ES cells or EBs in concentrations of RA at 0, 10, 100, and 1000 nM for 24 h. Subsequently, the experiments were repeated with solutions of RA released from microparticles allowing comparison of freshly prepared RA. ES cells and EBs were fixed in 3.5% paraformaldehyde for 20 min. Fixed cell samples were permeabilized in 0.1% (*w*/*v*) Triton X-100 (diluted in PBS) for 40 min at room temperature for cell monolayers and 90 min for cell aggregates. Following permeabilization, samples were covered in blocking solution for 30 min at room temperature. Aggregates were immunostained by incubation overnight at 4 °C with rabbit anti-Stra8 primary (Abcam) diluted 1:100. After washing 3 × 10 min with PBS, the aggregates were incubated for 2 h at room temperature with anti-rabbit Alexa Fluor 546 fluorescent secondary antibody (Invitrogen) diluted 1:200. Immunostaining was visualized by conventional fluorescence and confocal microscopy. In all experiments, qualitative observations are presented and described and for each individual experiment images were collected at similar exposure settings.

### Retinoic Acid (RA) Microparticle Encapsulation

Retinoic acid containing PLGA/TBIIF (70:30) polymer microparticles with an average size of 5 μm were produced using a single-emulsion, water-in-oil encapsulation method as previously described [19]. Briefly, 2 mg RA was solubilized in dichloromethane (DCM) along with 700 mg PLGA and 300 mg TBIIF, 10 mg FITC-BSA (or 10 mg unlabeled BSA), and 200 mL of 0.3% (*w*/*v*) poly vinyl alcohol and homogenized at 4000 rpm for 2 min. The solution was stirred at 300 rpm for 4 h to allow the DCM to evaporate. The resulting microparticles were then collected by centrifugation, the particles were washed with distilled water three times and snap frozen in liquid nitrogen and freeze dried (Edwards Modulyo D, IMA Edwards, UK) for 2 days.

### Encapsulation Efficiency of Microparticles

Using an adapted version of the method published by Sah (1997) [20], 15 mg of loaded PLGA microparticles was dissolved in 750 μL of DMSO and 2150 μL of 0.02% (*w*/*v*) SDS in 0.2 M NaOH for 1 h at room temperature and 150-μL aliquots of each solution were added to a well plate and a bicinchoninic acid (BCA) assay (Sigma-Aldrich) was performed. Appropriate standards of BSA were created, and after 2 h of incubation at 37 °C, the plate was scanned at 562 nm on a plate reader. The total protein content was then calculated via a polynomial equation of the standard curve, and the encapsulation efficiency was calculated from the theoretical expected loading of the microparticles. The microparticle batches were lyophilized and the powder was vacuum packed and stored at 4 °C until required. The size distribution of microparticle batches was determined by suspension in deionized water (20 mg/mL) and sized using a laser diffraction method (Coulter LS230; Beckman Coulter, UK).

For release kinetics, 25 mg of microparticles was suspended in 1.5 mL of PBS in glass tubes and then placed on a GyroTwister and gently rocked at 5 rpm at 37 °C. At 24-h intervals, the tubes were centrifuged at 3000 rpm for 3 min. The supernatant was then carefully removed and stored at – 20 °C for analysis. The microparticles were then re-suspended in 1.5 mL PBS and returned to the incubator; this process was repeated over 12 days to ensure complete release from the microparticles.

### Fluorescence-Associated Cell Sorting (FACs) or RA Microparticles

Microparticles containing FITC-BSA RA or BSA RA and 5-μm reference beads were suspended in PBS (25 mg/mL) and sonicated for 30 s to break up aggregates before being added to separate 5 mL FACS tubes under sterile conditions. Sorting and analysis were performed using a MoFlo Astrios Cell Sorter (Beckman Coulter, UK) equipped with a 488-nm laser at 100 mW of power. Forward scatter (FSC1) and side scatter (SSC) were collected through a filter and the FITC signal was collected in the FL1 channel through a 513/26 bandpass filter. A light scatter gate was drawn in the SSC versus FSC1 plot to include microparticles of a similar size to 5-μm reference beads. Cells within the gate were displayed within a SSC versus 488,513/26 intensity plot allowing a visualization of the fluorescence intensity distribution within the microparticle batches. A final selection gating was applied to sort based on fluorescence intensity. Microparticles were sorted over several sessions in separate batches to reduce the time spent suspended in PBS and were freeze dried for long-term storage.

### HOTs Patterning Procedures with Embryoid Bodies and Microparticles

The movement and positioning of cells and microparticles was as described previously [10]. Briefly, the system uses a Nd:YAG, solid state, infrared (1064 nm), 3 W maximum output, continuous wave, class 4 3.2-mm-beam-diameter laser adapted for biological applications (Laser Quantum). Optical manipulation with multiple optical traps is achieved by expanding the laser beam so that it overfills the aperture of the spatial light modulator (SLM) chip (512 × 512 pixel ferroelectric liquid crystal (FLC) array) (Laser 2000). This is then coupled with the optical tweezer system by imaging the SLM onto the back aperture of a high numerical aperture oil immersion microscope objective lens (40 × 1.3 NA Zeiss, Plan-NeoFluar). The resulting traps can then be focused anywhere within the field of view, with controlled holograms generated by the SLM giving full axial and lateral control over the trapping beam. All software-based control functions were programmed using LabVIEW software (National Instruments) as described previously [10]. A patterning, microfluidic-type gasket with multiple wells connected by channels was made (see supplementary material). This enabled EBs and microparticles, together with pre-GelMA solution, to be added to the wells and then moved and assembled by user-specified design with the HOTs. The GelMA was prepared as described previously [10] and then dissolved in 80 °C photo-initiator (Irgacure 2959 0.5% (*w*/*v*)) to yield a 10% (*w*/*v*) GelMA solution, and stored in the dark at 4 °C until use. This solution was warmed to 37 °C and then added to the patterning gasket to a maximum volume of 100 μL. Cells and microparticles to be patterned were added directly to the patterning gasket as required under sterile conditions. Once patterning was completed, the GelMA solution was cross-linked with a 5-s burst of UV light from a distance of 5 cm resulting in an output of 30 W/cm^2^ using a UV lamp (Omnicure S2000; JentonUV, UK). The GelMA was left for 5 min to ensure complete crosslinking. If prolonged cell culture was required, cell culture medium was added on top of the cross-linked GelMA before incubation at 37 °C with 5% CO_2_.

## Results

### Retinoic Acid Dose Response-Induced Expression of Stra8 in mES Cells

**Fig. 1:**
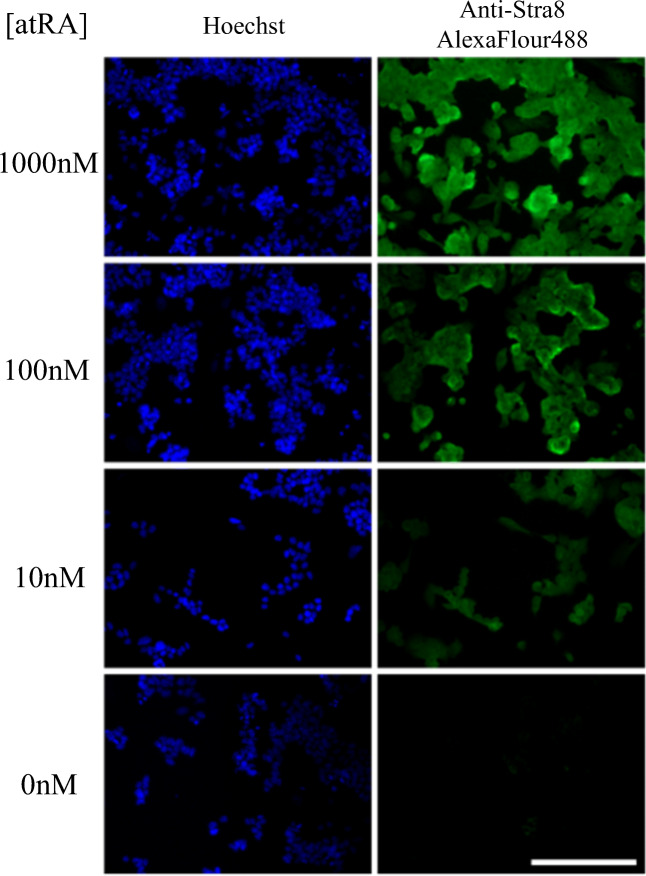
Dose–response effects of RA on Stra8 expression in mES cells. Stra8 immunocytochemistry staining showing the response of mES cells to a range (0–1000 nM) of RA concentrations. Cells were counterstained with Hoechst dye. Scale bar represents 100 μm. atRA, all *trans* retinoic acid

The dose response experiment in Fig. 1 shows induced expression of mES cells to a range of RA concentrations, from 0 to 1000 nM. The cells were serum starved for 24 h prior to being exposed to RA for 24 h. The fluorescence staining intensity gives an indication of the level Stra8 protein expression and is seen to increase with increasing RA concentrations. Little or no staining was seen in absence of added RA. This contrasts with the low-level staining seen without transient serum starvation (data not shown).

### Retinoic Acid Dose Response-Induced Expression of Stra8 in Mouse EBs

**Fig. 2:**
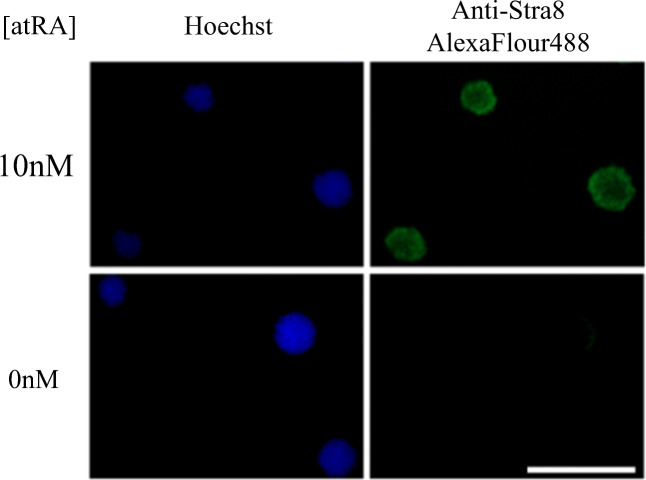
Induced expression of Stra8 by RA in mEBs. Stra8 immunostaining in mEBs in response to RA (10 nM), with Hoechst counterstaining. Scale bar represents 100 μm. atRA, all *trans* retinoic acid

To show effective delivery and induction of Stra8 in 3D cell aggregates, mouse EBs were exposed to either 0 or 10 nM RA for 24 h. Figure 2 shows clear staining and expression of Stra8 in response to 10 nM RA, with negligible staining seen in the absence of RA. Prior to aggregation, the ES cells were serum starved for 24 h, and this data indicates that there were no deleterious effects on EB formation or Stra8 gene expression and background Stra8 expression remains negligible.

### Retinoic Acid Encapsulation and Induction of Stra8 in mES Cells

**Fig. 3:**
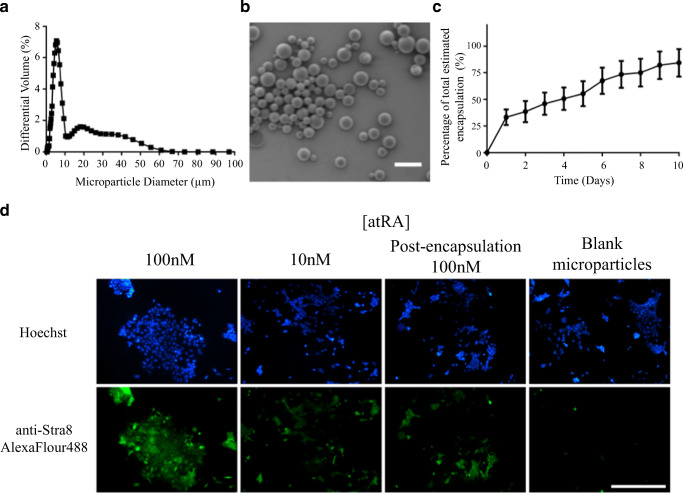
Characterization of RA-laden microparticles. **a** Microparticle size distribution by differential volume Coulter analysis. **b** SEM micrograph of the microparticles demonstrating their spherical, non-porous morphology. **c** In vitro release study over 10 days (error bars mark cumulative standard error of the mean, minimum of three replicates). **d** Representative immunofluorescence staining of Stra8 for each condition and bioactivity assessment comprising fresh solutions of 10 and 100 nM RA and solutions extracted from a suspension of RA-containing microparticles, estimated by microparticle mass and estimated encapsulation efficiency to contain 100 nM RA (post-encapsulation 100 nM). Scale bar represents 100 μm

The RA microparticles (BSA-RA co-loaded) had an average size of around 5 μm (Fig. 3a), and SEM imaging showed they had spherical, non-porous morphology (Fig. 5b). The in vitro release study (Fig. 5c) showed an initial burst release within the first 24 h, followed by a more gradual release over the next 10 days. The bioactivity assay (Fig. 3d) showed Stra8 staining in mES cells in response to freshly prepared RA at concentrations of 10 and 100 nM and a solution collected from a suspension of RA-containing microparticles, estimated by microparticle mass and estimated encapsulation efficiency, to have a RA concentration of 100 nM. It can be seen that the solution collected from RA encapsulated microparticles showed a level of staining somewhere between that seen with fresh RA at concentrations 10 and 100 nM and confirmed the ability to deliver RA from microparticles.

**Fig. 4:**
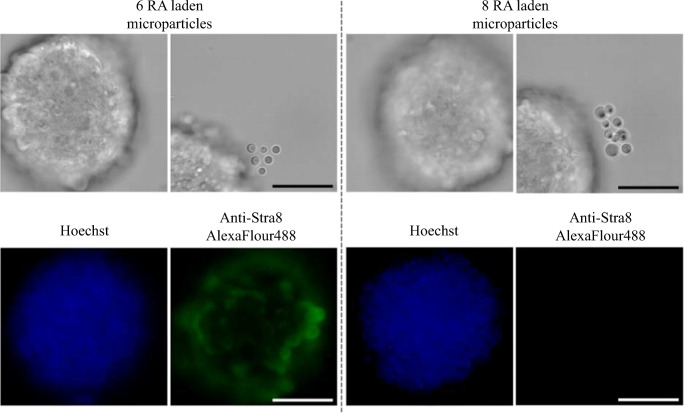
HOTs patterned RA microparticles for zonal stimulation of Stra8 in mES aggregates. **a** Bright-field micrographs showing the GelMA stabilized 6- and 8-microparticle patterns around mES cell aggregates with a HOTs patterning platform. **b** Fluorescence immunostaining for Stra8 with Hoechst counterstaining. Representative of *N* = 4 experiment repeats. Scale bars represent 50 μm

Figure 4 shows an example of the results of HOTs patterning experiments with a defined number of BSA-RA microparticles precisely positioned around mEBs. Immunostaining for Stra8 was only seen in approximately only 50% of experiments (*n* = 4) and was likely due to variability in loading efficiency in the microparticles. This data is included to show the evolution of the experiment and our work toward induction of a highly localized response in the EBs. The 6- and 8-microparticle patterns were arbitrarily chosen to produce an obvious, user-defined pattern and to achieve a localized or focused delivery source. There were no differences in observed effects between 6- and 8-microparticle patterns and the 6-microparticle triangular configuration was used in all subsequent experiments.

### Analysis and Sorting of the FITC Co-Loaded RA Microparticles

**Fig. 5:**
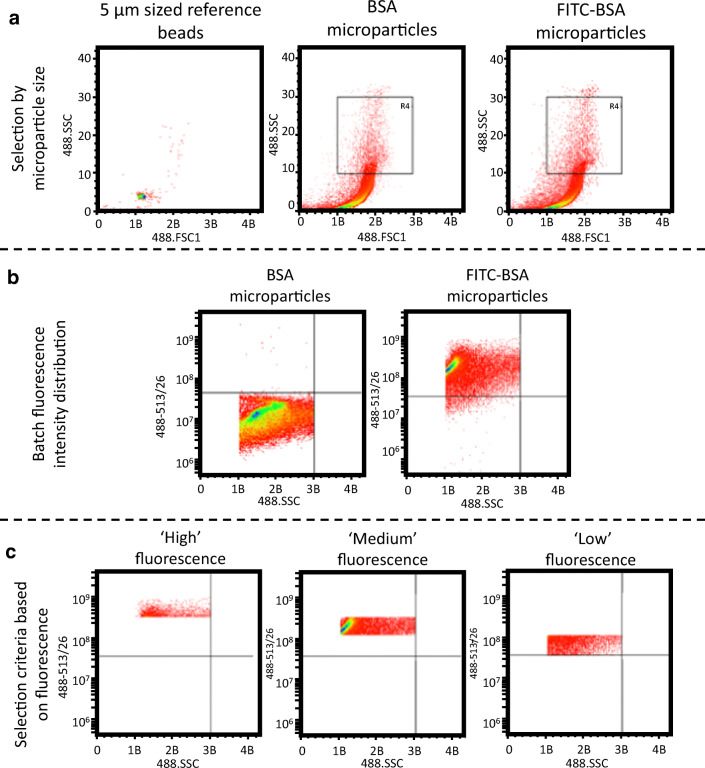
Analysis and sorting of the FITC co-loaded RA microparticles. FITC-BSA co-loaded RA microparticles were analyzed using FACS. A FACS analysis by means of SSC vs. FSC1 to gate for appropriately sized microparticles based on 5-μm reference beads. **b** The R4 gating plotted as SSC vs. fluorescent intensity (488–513/26 nm). **c** Representative sorting gating used to yield high, medium, and low fluorescent intensity microparticle groups

In order to be able to select small numbers of RA-laden microparticles and ensure high encapsulation efficiency of the selected microparticles, a method of co-loading FITC-BSA and RA was developed. Figure 5 shows analysis and selection of FITC co-loaded RA microparticles and sorting into different groups of microparticles with fluorescence intensity ranging from “High,” “Medium,” and “Low.” The microparticles were initially gated based on their size versus 5-μm reference beads, assessed by forward and side scatter (Fig. 5a). As a means of comparison, the non-fluorescent RA-laden microparticles were also analyzed to provide a baseline fluorescence intensity. The use of these microparticles as a control ensured that any RA or polymer autofluorescence would be normalized from the FITC-labeled batch. When comparing these two batches in the “Batch fluorescent intensity distribution” (Fig. 5b), it can be seen that the FITC co-loaded microparticles had a much greater fluorescence intensity. Three separate groups were created to select for “High,” “Medium,” and “Low” fluorescence microparticles, as shown in the representative plots (Fig. 5c). Then by running a high-density suspension of the FITC co-loaded batch through the FACS process, the three separate suspensions (“High,” “Medium,” and “Low”) and any sub-“Low” microparticles were separated.

### Analysis of the FACS Selected “High” Loading Microparticles

**Fig. 6:**
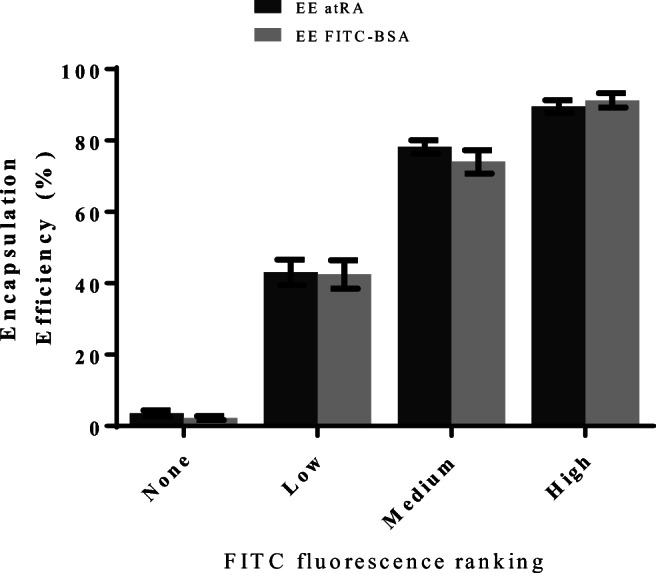
Post-sorting encapsulation analysis of the FITC-BSA co-loaded RA microparticles. A plot showing the estimated encapsulation efficiency by means of protein (FITC-BSA) and RA loading of the four sorted microparticle groups. Bars relate to the mean encapsulation efficiency with bars showing standard error of the mean. Minimum of 5 replicates and 3 separate experiments of Student’s *t* test analyses were performed, and the significant difference is indicated accordingly, ****p < 0.001, ***p < 0.0045, ***p < 0.01, and *p < 0.0253. EE, encapsulation efficiency

Figure 6 shows that FITC fluorescence correlates with encapsulation efficiency of BSA and by extension with RA. With increased FITC fluorescence, greater RA is estimated to be encapsulated within the batch. The “High” group was estimated to have an encapsulation efficiency of 89.5 ± 1.8 and collectively this was expected to achieve a robust and reliable selection and delivery process for the HOTs patterning experiments.

### HOTs Patterning of FITC-BSA/RA Microparticles Around mEBs

**Fig. 7:**
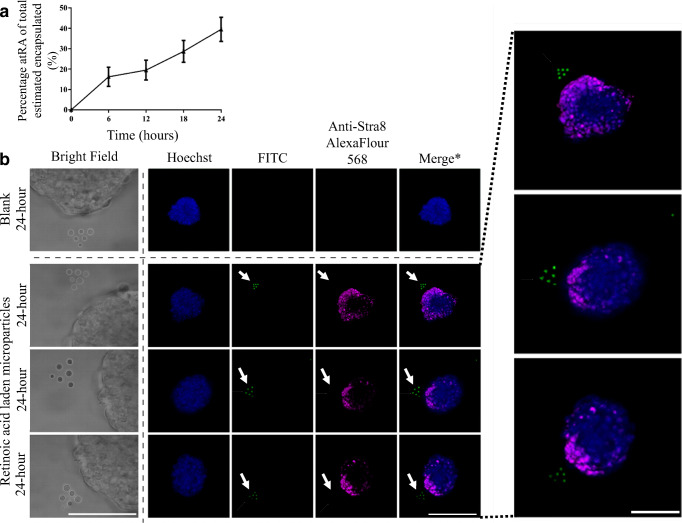
Zonal delivery of retinoic acid to mEBs. **a** Plot of the in vitro release study completed over 24 h for the release of retinoic acid from retinoic acid encapsulated microparticles. Error bars represent the cumulative standard error of the mean. **b** Fluorescence and confocal imaging showing the positioning of 6 retinoic-acid-laden FITC microparticles arranged into a triangular structure in close proximity of the mEBs with the HOTs patterning platform and immunostaining for Stra8 at discrete locations after 24 h. Representative of *N* = 5 experiment repeats. Scale bars represent 50 μm

Figure 7a shows the release of RA from the FITC-BSA co-loaded RA microparticles over 24 h. By the 24-h point, 39.5 ± 5.9% of the loaded RA was released. Figure 7b shows the positioning of six RA-releasing microparticles in a defined triangular pattern formed in close proximity (within 20 μm) to mEBs and stabilized by use of GelMA cross-linking. After 24 h, immunostaining for Stra8 can be clearly seen in localized regions within the EBs closely apposed with wherever the RA release source was placed around the EBs. Through the use of FACS sorted “High” loading RA microparticles, experimental success was raised from 50% (*n* = 4 separate experiments) to 80% (*n* = 5 separate experiments).

In this experiment, we focused entirely on positioning of the microparticles at defined locations around EBs and inducing highly localized responses while maintaining the same distance that the microparticles were positioned from the EBs at each set location. In a related study, we have investigated the ability to control specific cellular responses over defined distances using a model of chemotactic responses of mouse osteoblasts (see supplementary material).

## Discussion

In this study, we have shown that we can build 3D cell microenvironments with defined, reproducible control and precision delivery, with micron resolution, of a biochemical signal gradient and to induce a highly localized biological response in multicellular aggregates directly in response to the biochemical signal.

The spatial and temporal induction of Stra8 in mEBs in response to RA [17, 18] released from microparticles positioned at discrete locations around the 3D cellular structures has served primarily as exemplar of the HOTs technology to precisely deliver and induce a biological response. This builds on our previous work with HOTs on assembly of complex microenvironments [10] to show that the technology is also very amenable to creating biochemical gradients. It also shows how the HOTs technology can complement other technologies on cellular assembly, as we have shown here by precision engineering of the microenvironment around EBs formed by the hanging drop method [13]. We envisage that this can be applied to organoids and organ-on-chip models and collectively can bridge gaps with the scale of cellular assembly, precision of cellular organization and accurate control of delivery of bio-instructive signals [1–8].

The sensitivity and relative simplicity of the RA-inducible Stra8 model [17, 18] was extremely useful for refining the delivery of biochemical factors from microparticles and specifically adapting their use for selecting and positioning small numbers of microparticles. Formulation of microparticles and the controlled release of soluble biochemical factors and drug molecules is well established with wide-ranging, demonstrated applications [21–28]. In many cases, controlled release studies are performed with bulk volumes of microparticles, and while it is known that loading within individual microparticles can be highly variable, this is relatively unimportant unless there is a need to work with individual microparticles. As we found in our studies with HOTs positioning of RA microparticles, in some experiments Stra8 expression was induced in EBs, and in other experiments, no expression was seen, suggesting variable loading of RA in the HOTs selected microparticles.

We were able to overcome this limitation by co-loading microparticles with FITC-BSA and RA and through FACS we were able to specifically select particles with high loading efficiency and markedly increase the success rate in inducing localized expression of Stra8. The burst release kinetics of the microparticles, combined with the ability to reproducibly and accurately position them at defined locations around the EBs, stabilized with GelMA, facilitated highly localized RA-induced responses in regions of the EBs directly facing the RA microparticles. The choice of pattern of 6 microparticles, roughly in an equilateral triangle configuration with the “base of the triangle” facing the EBs, was an arbitrary choice intended to clearly show that it was precisely patterned and included a sufficient number of microparticles to achieve localized, focused release or RA toward the EBs sitting in close apposition. As we have previously shown, the HOTs technology offers considerable scope and flexibility to create a wide range of defined patterns with microparticles, scaffolds, and cells [10].

Although we have not quantified the induced, localized expression of Stra8, the response is highly defined and striking and serves to demonstrate a key objective of this study. In related work, we have demonstrated and quantified chemotaxis of mouse primary calvarial osteoblasts to platelet-derived growth factor-BB (PDGF-BB), a known potent chemotactic factor for osteoblasts. Agarose beads doped with PDGF-BB (10 nM) and single mouse primary calvarial osteoblasts were positioned at defined distances apart (50, 100, and 150 μm) using HOTs and stabilized within gelMA (see supplementary material). As expected, there was very clear movement of the osteoblast toward the PDGF-BB-doped bead, with a trend of decreasing distance moved with increasing distance of separation. Over the 8-h time course of this experiment, it would have been reasonable to expect a more marked gradation of chemotaxis but, overall, there was no significant difference in net positive migration relative to distance from the release source. These observations serve to highlight the challenges of creating and quantifying biochemical gradients and the many variables involved, including the properties of the biochemical factor, the kinetics of release, and the properties of the environment through which the factor is distributed. What is absolute from this study is that the HOTs technology and the approach described here is highly amenable for precision assembly of tunable, complex microenvironments with spatial and temporal control of both cellular organization and delivery of bio-instructive signals.

In summary, we have demonstrated the creation of biochemical gradients within 3D cellular microenvironments and the precision delivery of bio-instructive signals by interactively positioning biochemical-laden microparticles around multicellular 3D aggregates at user-defined locations. While challenges remain, such as quantifying signal gradients within cellular microenvironments, this robust, highly interactive “cause-and-effect” type model is able to accurately target and provide insights into understanding and quantifying specific biological responses in 2D or 3D cell models.

## Conclusion

The construction and interrogation of multicellular 3D models and microenvironments and the capability to recapitulate complex physiological and pathophysiological processes in vitro is being advanced significantly by various innovative techniques and technologies, yet our understanding remains far from complete.

Using HOTs, we can assemble complex microenvironments and create biochemical gradients with a level of control from micron- to millimeter-length scales. As we have also shown, this technology is very adaptable and can be used with other technologies such as organoids and microfluidics. Collectively, this shows that we can create more accurate in vitro representations of the native tissue structures and signaling interactions and to build multicellular 3D cell models that enable more detailed in vitro investigation and interrogation of physiological and pathophysiological responses.

## Electronic supplementary material

ESM 1 (DOCX 785 kb)
