# Supplementary material for: Localized Induction of Gene Expression in Embryonic Stem Cell Aggregates Using Holographic Optical Tweezers to Create Biochemical Gradients
Source: Regen Eng Transl Med. 2019 Aug 26;6(3):251–61. doi: 10.1007/s40883-019-00114-5 (PMC7505830; doi:10.1007/s40883-019-00114-5)
Supplement: Supplementary file 1 — (DOCX 785 kb) [file 40883_2019_114_MOESM1_ESM.docx]

**Localized induction of gene expression in embryonic stem cell aggregates using holographic optical tweezers to create biochemical gradients**

**Kirkham et al,**

Supplementary data

To create the lobed patterning gasket, an internal mould was made for the gasket to be cast around it. The internal mould was designed using a simple open source Computer Aided Design (CAD) approach (Tinkercad). The design was then 3D printed with polylactic acid (PLA) filament using a MakerBot Replicator 2. The printed internal mould was then glued to a 60 mm petri dish (using a non-toxic silicone rubber compound). Sylgard 184 silicone elastomer was prepared as described previously and was left to degas for 4 hours at room temperature. The degassed solution was then carefully added to the internal-mould-containing petri dish up to a depth of 5mm so as not to cover the mould and to create a suitable volume patterning gasket. The petri dish was covered with its corresponding lid and left for 5 days at room temperature to fully cure. Once Chapter 4 Results II 103 cured, the PDMS was removed from the petri dish and cut to size to leave the internal-mould-shaped area surrounded my PDMS. The PDMS lobed patterning gasket is then glued to polyHEMA-coated coverslip glass and stored at room temperature until required. Before use in cell patterning, the PDMS lobed patterning gasket was UV sterilised for 1 hour.


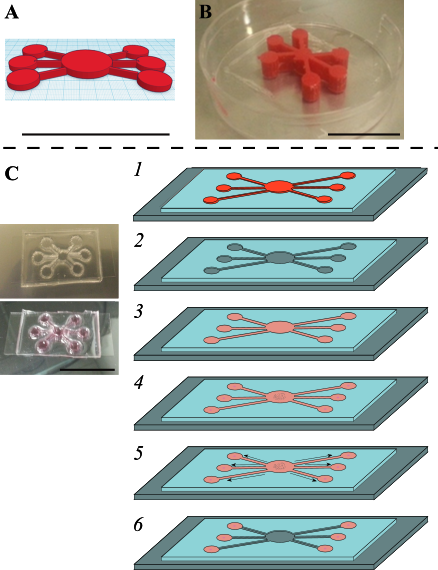


Figure S1. The design and approach for patterning with the lobed patterning gasket. How the lobed patterning gasket was designed (A), fabricated (B) and used (C). A. Internal mould design created with Tinkercad. B. 3D-printed PLA mould. C. Steps taken to use the lobed patterning gasket: 1) in a petri dish (see B), the PDMS gasket was formed around the internal mould; 2) the PDMS gasket was removed from the petri dish and glued to a glass coverslip; 3) the lobed patterning gasket was filled with pre-gelled hydrogel; 4) release sources were added to the central reservoir and cells to the outer lobes; 5) individual release sources were then translocated as required via optical manipulation to each cell-containing lobe and patterned; 6) the hydrogel was crosslinked and the central reservoir and channels excised. The scale bars represent 20 mm.


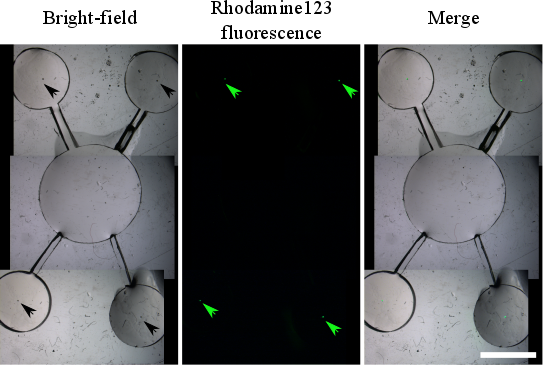


Figure S2. Demonstration of the four-lobed patterning gasket. A bright-field and fluorescence micrograph of the four-lobed gasket after individual rhodamine-123-laden microparticles (Indicated by arrows) were translocated and isolated in the outer lobes of crosslinked

Osteoblasts are known to demonstrate chemotaxis to a range of biochemical factors, with PDGF-BB known to exert a potent chemotactic response (1). We have worked extensively with mouse primary calvarial osteoblasts and using these cells, isolated as described previously (2) we have investigated chemotaxis of these cells to PDGF-BB at different stages of osteogenic differentiation *in vitro*. Using the IBIDI μ-Slide Chemotaxis 2D (Thistle Scientific, UK) system we have demonstrated and quantified potent chemotactic responses to PDGF-BB over a range of concentrations and established 10 nM PDGF-BB to induce a marked positive chemotaxis response (data not shown). Time-lapse images were collected every 15 minutes and experimental data assessed in ImageJ (NIH) and the MTrackJ plugin was used to manually track the cells.

Using this approach we devised an experiment to quantify chemotaxis of individual mouse primary osteoblasts to a HOTs-positioned PDGF-BB-soaked agarose bead placed at defined distances from the osteoblast. Agarose beads have been used quite extensively in developmental biology studies to deliver biochemical signals and provides a method to deliver, quite accurately, an amount of a biochemical factor from a single release source. Here, we used 4% (w/v) agarose beads, which were approximately 50 microns in diameter. The beads also contained 10% bovine serum albumin, which helped provide contrast between the bead and the aqueous environment for HOTs trapping (reference 10 in main body of the paper). Agarose beads were soaked at 4^0^C overnight in 10 nM PDGF-BB or 5mg/ml α-chymotrypsin which was used as a negative control / model release protein.

Using the HOTs platform as described in the main body of the paper, single agarose beads and single mouse primary osteoblasts were positioned in-line with each other and separated over distances of 50, 100 or 150 microns. The position of the bead and osteoblast was stabilized in 10% gelMA as described in the main body of the paper. Images were taken every 15 minutes over an 8 hour time course and net migration recorded as described above. From the representative micrographs and overlaid tracks, it is clear that the PDGF-BB-soaked beads are directing chemotaxis of the osteoblasts towards them, with no response seen with α-chymotrypsin control beads.


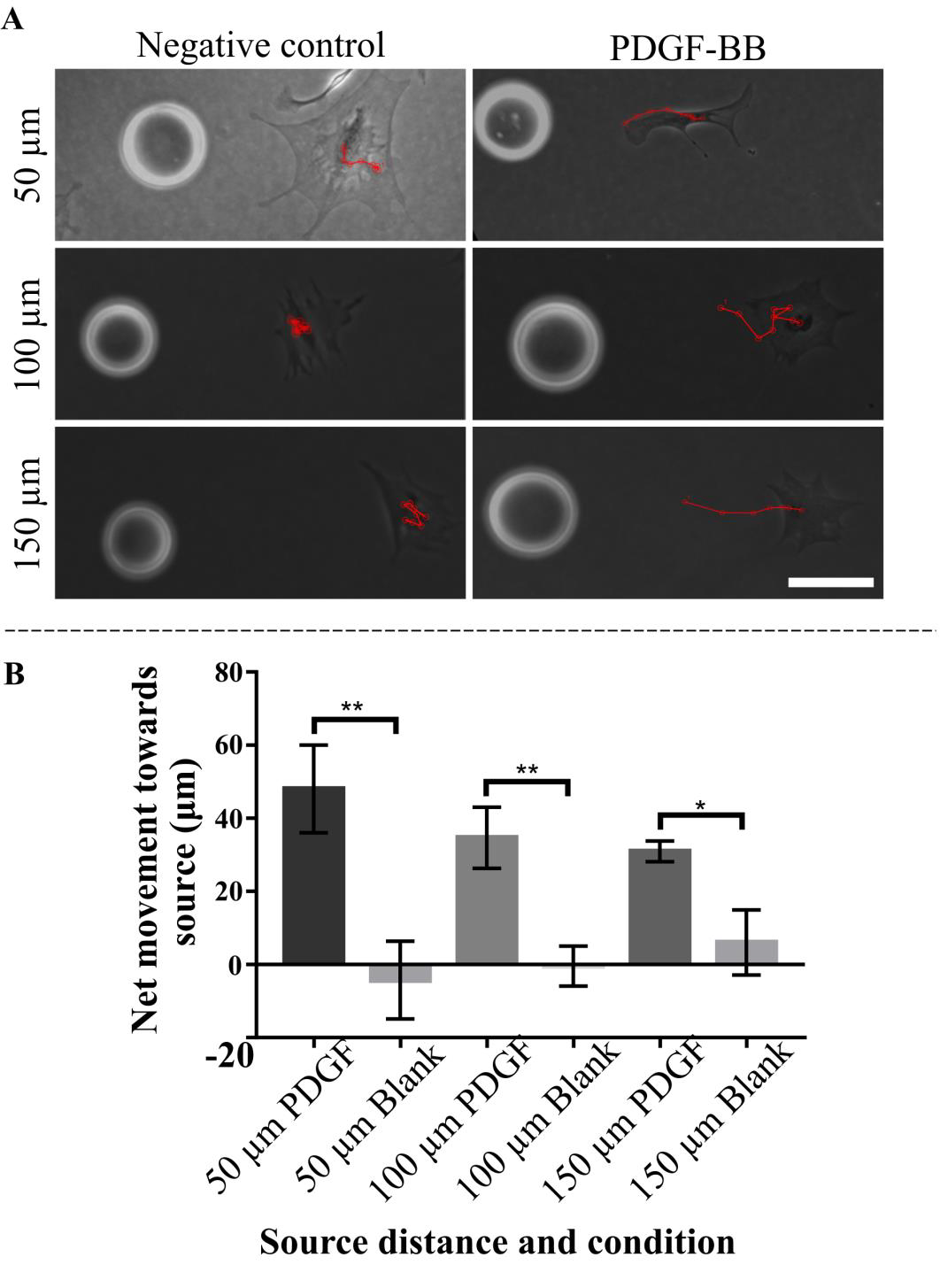


Fig S3. Images of PDGF-BB- (10 nM) or α-chymotrypsin- (5mg/ml; ‘Negative control’) -soaked 4% (w/v) agarose beads and their effect on mPC migration (A). The red track shows the movement of the mPC cell from the imaged point of origin. Scale bar represents 50 μm.

A plot displaying the average net migration with respect to the agarose bead release source. Error bars display the standard error of the mean (B). Data based on 10 replicates and 3 experiments. Statistical comparisons were done by Students t test, ** P<0.0045 and * P<0.0253.

1. Sanchez-Fernandez, M. A., Gallois, A., Riedl, T., Jurdic, P. & Hoflack, B. Osteoclasts control osteoblast chemotaxis via PDGF-BB/PDGF receptor beta signaling. *PLoS One* **3,** e3537 (2008).
2. Sidney LE, Kirkham GR, Buttery LD. [Comparison of Osteogenic Differentiation of Embryonic Stem Cells and Primary Osteoblasts Revealed by Responses to IL-1β, TNF-α, and IFN-γ](javascript:void(0)). Stem cells and development 2014; 23, 605-617
